# Inflammatory Molecules Responsible for Length Shortening and Preterm Birth

**DOI:** 10.3390/cells12020209

**Published:** 2023-01-04

**Authors:** Zacharias Fasoulakis, Antonios Koutras, Thomas Ntounis, Panos Antsaklis, Marianna Theodora, Asimina Valsamaki, George Daskalakis, Emmanuel N. Kontomanolis

**Affiliations:** 1 1st Department of Obstetrics and Gynecology, National and Kapodistrian University of Athens, General Hospital Alexandra, 11528 Athens, Greece; 2 Department of Internal Medicine, Koutlimbaneio and Triantafylleio General Hospital of Larissa, 41221 Larissa, Greece; 3 Department of Obstetrics and Gynecology, Democritus University of Thrace, 68100 Alexandroupolis, Greece

**Keywords:** preterm birth, cervical insufficiency, inflammation, interleukins, chorioamnionitis

## Abstract

It is estimated that inflammation at the placental–maternal interface is directly responsible for or contributes to the development of 50% of all premature deliveries. Chorioamnionitis, also known as the premature rupture of the amniotic membrane in the mother, is the root cause of persistent inflammation that preterm newborns experience. Beyond contributing to the onset of early labor, inflammation is a critical element in advancing several conditions in neonates, including necrotizing enterocolitis, retinopathy of prematurity, bronchopulmonary dysplasia, intraventricular hemorrhage, retinopathy of prematurity and periventricular leukomalacia. Notably, the immune systems of preterm infants are not fully developed; immune defense mechanisms and immunosuppression (tolerance) have a delicate balance that is easily upset in this patient category. As a result, premature infants are exposed to different antigens from elements such as hospital-specific microbes, artificial devices, medications, food antigens and hypoxia/hyperoxia. This has detrimental implications for preterm deliveries of less than 28 weeks because they have not yet evolved the mechanisms to tolerate maternal and self-antigens.

## 1. Introduction

There is a correlation between rising intrauterine infections and 40% of spontaneous preterm births (sPTB). Typically, the cervix prevents infections from entering the amniotic sac by acting as a physical and immunological barrier between the vagina and the amniotic sac. However, changes in the cervix occur during pregnancy that alter these mechanisms. It remains closed and firm from the onset of pregnancy until the end of the third trimester. As the fourth trimester progresses, the cervix begins to efface and enlarge in preparation for the infant’s birth [1]. These modifications to the cervix take place promptly and appropriately up until the mother is ready to give birth. However, the presence of risk factors such as acute and persistent infection and local inflammation in the cervix can cause damage to the integrity of the cervix, leading to premature remodeling and, ultimately, sPTB. In earlier clinical investigations, infections with bacteria (such as chlamydia, gonorrhea, mycoplasma, etc.) and viruses (such as herpesviruses and human papillomaviruses) have been linked to an elevated risk of PTB. Despite this, the particular cause of PTB is not completely understood [2]. 

The well-being of neonates is negatively impacted in the short and long term by preterm birth, defined as occurring before gestation reaches 37 weeks by the World Health Organization. Premature membrane rupture and labor account for over 70% of all preterm deliveries. In almost half of cases, chorioamnionitis and maternal infection are associated with premature birth. In light of the extent of these problems, this review aims to investigate the correlational factors that contribute to premature birth and inflammation [3,4].

## 2. Preterm Birth and Immune Changes

As elucidated in the preamble, PTB pertains to birth that occurs before 37 weeks of gestation, a definition that the World Health Organization has endorsed. Premature births accounted for 11.1% of all deliveries in 2010, accounting for 14.9 million births worldwide. Preterm birth accounts for about 5% of births in European countries and approximately 18% in African countries. Children born prematurely have an augmented risk of mortality before their fifth year of life. The financial burden of neonatal intensive care is substantial, and its emotional toll on families can last for years [1,5].

The deposition of paternal antigens on fetal tissues relies on feto-maternal immunological tolerance, as shown in a number of studies. The uterus is invaded by fetal trophoblast cells. During first contact with seminal fluid during copulation, the mother’s immune system recognizes and reacts to fetal or paternal antigens; this process is repeated several times throughout pregnancy. By stimulating the production of proinflammatory cytokines and the recruitment of leukocytes to the uterine lining, the inclusion of cytokines, chemokines and prostaglandins in seminal fluid makes implantation itself an inflamed process. This inflammation must subside, and a tolerogenic environment must be developed around the time of implantation [5,6]. 

Among the many processes set in motion to create this tolerogenic setting are the release of anti-inflammatory chemicals such as TGF and the generation of specialist anti-inflammatory T cells designated as CD4+FOXP3+ regulatory T (Treg) cells, which restrain anti-fetal inflammatory immune responses [7,8,9]. Tolerogenic dendritic cells (DCs), which are only found in the decidua, play a crucial role in forming regulatory T cells (Tregs) by cross-presenting fetal antigens to maternal CD4+ T cells. In a two-way street of immunomodulation, regulatory T cells (Tregs) engage with dendritic cells (DCs) and macrophages (M) to induce tolerogenic phenotypes in both cell types. By preventing T effector (Teff) cell responses and maintaining anergy in the pool of T conventional (Tcon) cells that would otherwise grow into Teff cells, Treg cells play a vital role in immunological defense against anti-fetal reactions. Furthermore, placental cells contribute to a tolerogenic microenvironment by increasing operationally repressive CD4+FOXP3+ Treg cells and restricting the stimulation of T helper (Th)1-, Th17- and Th2cytokine-producing Teff cells [10,11,12]. These cytokines are released by placental cells in addition to IL-10 and trophoblast-derived colony-stimulating factor (CSF)1 (formerly M-CSF) [7,8,9,10,11,12,13,14,15,16].

## 3. Immuno-Inflammation and Normal Delivery

Because the maternal and fetal circulatory systems interact in the placenta, there is a significant amount of interaction between them during a normal pregnancy. In addition to nutrients and removing waste products, a variety of additional substances, including cells, signaling molecules, extracellular vesicles (ECVs) and nucleic acids, are exchanged between the fetus and mother. Microchimerism occurs when cells are transferred between the mother and the developing child; when cells from the mother are given to the fetus, this is called maternal microchimerism (MMC), and the transfer from the fetus to the mother is dubbed as fetal microchimerism cells (FMC) [17]. Research has demonstrated that the effects of MMC and FMC can persist in many tissues and organs for decades after pregnancy, including the brain, bone marrow, spleen, skin, heart, lungs, and lymph nodes [18,19]. 

Maternal microenvironment (MEC) cells induce NIMA-specific CD4+FOXP3+ Treg cells in fetal immune cells to enhance tolerance to maternal antigens throughout pregnancy. In addition, if they can keep their NIMA tolerance up through the generations, it could help future offspring be more fertile. Despite its usefulness, this information is largely derived from a mouse model and is restricted to situations in which paternal antigens and NIMA were swapped [20,21]. The placenta, microvesicles and exosomes all play roles in the transport of cell-free (cf) proteins and nucleic acids such as cfRNA and cfDNA. Near the end of pregnancy, there is an increase in the amount of fetal cell-free DNA (cfDNA) and RNA transcripts in the mother’s blood. Apoptotic fetal cells are a source for fetal cfDNA and RNA, and CD71+ fetal erythroid cells are a rich source of fetal DNA in the mother’s blood. Both passive and active transmission of CfDNA and RNA transcripts by external cephalic versions (ECVs) have been reported [22,23,24].

One form of ECV is exosomes, which are discharged into the extracellular environment via the exocytotic pathway. Notably, the placenta is responsible for releasing exosomes into the mother’s bloodstream, with the number of exosomes in the mother’s bloodstream increasing with gestational age and reaching a peak at term [25]. Although the precise role of ECVs throughout pregnancy is unclear, it is thought that they facilitate fetal–maternal communication during pivotal events, including implantation, labor and delivery. Human villous trophoblasts, which have important regulatory roles in immunological signaling, produce exosomes that contain placenta-specific microRNAs (miRNAs) in the maternal blood. Exosomes originating from the mother are likewise increased in number during pregnancy, and there is a two-way exchange of exosomes between the mother and the developing fetus [26,27,28,29,30,31].

## 4. Immuno-Inflammation and Preterm Birth

What sets apart term and preterm labor could be an early imbalance of decidual inflammatory signals or a powerful aberrant stimulation (internal or external) that initiates inflammatory pathways. Anti-inflammatory mediators (including IL-10 and IL-4), in contrast to proinflammatory mediators (IL-1, IL-6, IL-8, TNF- and INF-), are downregulated in PTB [32,33]. In actuality, IL-10 has a major impact on preterm birth; it is generally thought of as a cytokine that helps keep the neonate inside the uterus. The symptoms of PTB syndrome are placental malfunction, early uterine contractions, membrane rupture and cervical dilatation. Additionally, myometrial, cervical, endometrial, decidual and placental pathology have all been linked to PTB [34,35]. Placental lesions indicative of maternal vascular under-perfusion are seen in approximately 30% of patients with PTB. Therefore, PTB may be considered a disorder similar to preeclampsia induced by defective deep placentation [36,37].

Furthermore, how a mother handles stress, infections and her diet impacts her developing child’s immune system, leading to impaired immune tolerance and an inflammatory response. Bacterial flora in the placenta is similar to that found in the mouth rather than the vagina. Inflammation and infection have been tied to as much as one-fourth of all preterm births. The unique triple “I” approach, which represents intrauterine inflammation, infection or both, emphasizes the fact that intrauterine inflammation can manifest itself in the absence of overtly harmful intrauterine infection. Furthermore, women with a short cervix are more likely to experience sterile intra-amniotic inflammation (10%) than microbial-associated intra-amniotic inflammation [38]. Despite this, both trigger the same cytokine mediators. It has been found that IL-6 and IL-1β are the two most essential uterine mediators throughout the transition period. High amounts of IL-6 are present at the start of labor, suggesting that it plays a role in implantation, pregnancy and birth. It is also implicated in stimulating amnion and decidual cells, culminating in increased prostaglandin production [39,40]. 

In addition, IL-6 in vaginal fluid has been extensively studied as a diagnostic biomarker of preterm delivery. There is a correlation between IL-6 levels, a marker of inflammation, and perinatal death and morbidity [36,39]. However, amniotic fluid IL-6 as a diagnostic marker for intraamniotic inflammation has been criticized by some researchers, who contend that this method fails to capture the full scope of intrauterine inflammation. Nonetheless, IL-1β affects the regulation of genes involved in inflammation and labor in the uterus. Furthermore, IL-1 promotes progesterone (P4) withdrawal by elevating nuclear progesterone receptor A, and it is a powerful activator of prostaglandin production by inducing COX2 [35]. In addition, IL-1-related pathways are elevated throughout the third trimester of pregnancy in women who give birth prematurely [36,39].

PTB is linked to increased choriodecidual inflammation, as well as increased M1 macrophages and NK cells when viewed from the scope of cells. PAMPs (infectious stimuli) or DAMPs (sterile stimuli) are two possible ways that a detrimental state can activate the innate system. The stress-exposed uterus, the developing fetus or the aging placenta can all release DAMPs. Some researchers have revealed that cfDNA triggers sterile inflammation and that fetal membrane aging is a crucial indicator at the commencement of labor. As a result of this innate stimulation, TLRs are activated, which in turn activate the production of proinflammatory cytokines, chemokines and leukocytes, ultimately resulting in the initiation of labor. Spontaneous PTB occurs if the deregulation of decidual inflammatory signaling occurs at an early stage. Mid-trimester endometrium inflammation, triggered by factors such as intrauterine infection or placental abruption, has also been linked to PTB. Moreover, smoking, anemia, a short cervix, a history of genital tract infections, racial/ethnic background and low or high birth weight may all be indicators of a lack of protective immunity inside the uterine tissues [35,36].

Genetic predisposition has been linked to PTB because it clusters in families, is highly heritable, can be identified through genetic susceptibility markers and reveals racial disparities. Women born prematurely have an increased risk of having premature kids in the future. Furthermore, at least one-third of PTB can be attributed to genetics, according to twin studies. An examination of maternal data revealed an upregulation of innate immunity-related genes and a downregulation of adaptive immunity-related genes, among the 210 genes shown to be differently expressed in PTB. Among these genes, 18 showed trimester-specific expression differences (mostly during the second trimester). It was discovered that the immune-related proteins IL-1R1 and tissue factor pathway inhibitor are differentially expressed and released longitudinally [37,38].

As a result, PTB has a polygenic foundation, meaning that it is caused by uncommon mutations or harmful variations in several genes associated with innate immunity and host defense systems against microorganisms and their toxic materials. Thus, inflammatory genes are activated and accelerate these processes. Furthermore, there are inherited polymorphisms that modify the inflammatory response in PTB, and there are identity genetic variations that alter the inflammatory response in PTB. TLR5, for example, is increased in the mother but downregulated in the fetus, a finding that is consistent with other recent studies. Epigenetic changes or variations due to incorrect fetal programming have also been linked to adult-onset illnesses in preterm infants [39,40,41,42,43]. 

Hormones that stimulate labor and reproduction inflammatory responses during pregnancy are aided by gestational hormones. For example, estrogen suppresses proinflammatory cytokines (IL-1; TNF-α; IFN-γ) and promotes anti-inflammatory IL-10, IL-4 and TGF-β. Nonetheless, IL-10 has been regarded as part of a pro-labor inflammatory response, playing a role in labor that varies with the tissue involved and can be either active or tolerant. Anti-inflammatory progesterone also helps stop uterine contractions and shields the developing fetus from harm. However, peripheral progesterone levels in humans are remarkable in that they do not fluctuate during pregnancy and only decrease after the baby and placenta have been delivered. In the last weeks of pregnancy, serum P4 levels rise, but the hormone’s ability to sustain a pregnancy weakens. This suggests that variations in labor may be attributable to the expression of nPRs (nuclear progesterone receptors) A and B. The primary receptor, nPRB, has anti-inflammatory properties that delay or stop labor and delivery [44,45,46].

Conversely, nPRB levels are higher than nPRB levels, with the former’s proinflammatory activities and the latter’s inhibition of nPRB activity, in the hours leading up to labor. A high ratio of nPRA to nPRB in myometrial cells indicates a proinflammatory state. Because proinflammatory or pro-labor stimuli trigger nPRA-mediated modifications, and because the main mechanism by which P4 is thought to halt labor is by reducing uterine cell reactivity to such stimuli, it is plausible to conclude that the inflammatory response is involved in labor induction. TLR4 expression is high but TLR2 expression is low, suggesting that P4 inhibits the immunological response to infection in PTB [47,48,49].

Therefore, it emerges that external P4 therapy prevents PTB by reducing maternal inflammation in the decidua and the cervix as well as by reducing the expression of contraction-associated proteins, inflammatory cytokines and slowing cervical ripening. Thus, PTB necessitates an inflammatory stimulation adequate to counteract the effects of progesterone. Intrauterine infection is a potent pathogenic driver for PTB, especially in the first several months of pregnancy. Later in pregnancy, when the inflammatory load on the mother is greater, P4 effects are attenuated, and tissue remodeling begins [50,51,52].

Many external modulators of the immune system have been previously accused of inflammation triggering and preterm delivery. Some of these have been widely studied, and there is much data both in animal models and in pregnant women. Air pollution and especially particulate matter (PM) with a diameter of 2.5 μm or less (PM2.5) can cross the blood–placental barrier, causing adverse perinatal effects, including miscarriage, intrauterine growth restriction, preterm birth and low birth weight [53]. Other factors commonly affecting the immune system are smoking, radiation, stress, working with pesticides, exposure to specific metals, inhaling anesthetics, and organic solvents, which are all factors associated with placental–maternal problems causing low birth weight, intrauterine growth restriction, preterm delivery as well as having long term effects on neuronal and behavioral development in neonatal and adult life [54].

## 5. Conclusions

Preterm delivery remains a serious public health issue. Inflammation represents a major pattern related to several factors essential to triggering labor. However, presenting earlier in pregnancy, it increases the risk of fetal damage, especially in the brain and lungs. Infants at risk for lung and brain abnormalities are identified by amniotic fluid evidence of inflammatory mediators. Postponing PTB and enhancing the outcomes of the neonates have proven to be quite difficult but of great importance. Vaginal progesterone therapy is currently one of the most administered therapeutic choices, however, with high rates of unsuccessful results. Furthermore, treating intrauterine infections with antibiotics has the potential to be detrimental to the growing fetus, raising the risk for cerebral palsy. Currently, therapeutic medicines that might target intrauterine inflammation with the goal of preventing fetal harm are being investigated in animal models. To lower the rates of fetal mortality and morbidity as a consequence of this clinical condition, it may be necessary to provide more individualized medical care.
